# α-Galactosylceramide-expanded virtual memory CD8^+^ T cells confer protection against a broad range of pathogens

**DOI:** 10.3389/fimmu.2026.1799271

**Published:** 2026-05-22

**Authors:** Jia-Xun Xie, Dong-Lin Li, Meng-Chih Lai, Yu-Ling Su, Chi Tai, Ching-Chun Wang, Jr-Shiuan Lin

**Affiliations:** Graduate Institute of Immunology, National Taiwan University College of Medicine, Taipei, Taiwan

**Keywords:** enteric infection, IFN-γ, IL-4, pulmonary infection, systemic infection, TNF-α, virtual memory CD8^+^ T cells, α-Galactosylceramide

## Abstract

α-Galactosylceramide (α-GalCer) treatment of animals is a well-established model to investigate the beneficial roles of invariant natural killer T (iNKT) cells in anti-infection and anti-tumor immunity. We previously found that α-GalCer treatment expands not only iNKT cells but also a group of antigen-inexperienced memory-like innate virtual memory CD8^+^ T (CD8^+^ T_VM_) cells. With the bystander protective potential of CD8^+^ T_VM_ cells, their roles in α-GalCer-mediated immunity against infection remain largely unexplored. Here, we reported that α-GalCer treatment conferred protection against systemic, enteric, and pulmonary bacterial infections, as well as pulmonary viral infection. This protection persisted after iNKT cells had diminished and coincided with sustained expansion of CD8^+^ T_VM_ cells. Adoptive transfer of α-GalCer-expanded CD8^+^ T_VM_ cells one day before infection significantly decreased bacterial and viral burdens. Mechanistically, α-GalCer treatment activated iNKT cells to produce IL-4, which subsequently expanded the number of CD8^+^ T_VM_ cells that were capable of producing TNF-α and IFN-γ. But the ability of each individual CD8^+^ T_VM_ cell to produce these cytokines was not enhanced. Lung-localized CD8^+^ T_VM_ cells were the primary contributors to α-GalCer-expanded CD8^+^ T_VM_ cells in the lung. α-GalCer-expanded CD8^+^ T_VM_ cells can reduce pulmonary viral titer in the host in a TNF-α-dependent manner in the absence of IFN-γ signaling. Together, these findings describe an immune cascade in which α-GalCer-activated iNKT cells orchestrate the expansion and function of CD8^+^ T_VM_ cells through IL-4 production, reveal previously unrecognized roles of CD8^+^ T_VM_ cells as downstream effectors in α-GalCer-mediated immunotherapeutic effects, and highlight the protective potential of CD8^+^ T_VM_ cells in host defense against diverse pathogens, thus providing a novel approach to developing new therapeutics for combating not only various infectious diseases but also tumors.

## Introduction

α-Galactosylceramide (α-GalCer), a marine sponge-derived glycolipid, is a natural ligand of invariant natural killer T (iNKT) cells and is commonly used to specifically activate iNKT cells through the presentation by MHC-class I-like molecule CD1d on antigen-presenting cells (APCs) (1). Upon α-GalCer stimulation, iNKT cells can rapidly proliferate (2) and produce a broad range of T_H_1, T_H_2, and T_H_17 cytokines (3), which subsequently activate dendritic cells (DCs), natural killer (NK) cells, and conventional T and B cells (4–7). These unique features enable iNKT cells to play an important role in bridging innate and adaptive immunity. Due to these immunomodulatory properties, α-GalCer has been extensively used to boost immune responses in different scenarios. Activation of iNKT cells by α-GalCer has been reported to have anti-tumor functions and has been used in clinical trials (8, 9). Additionally, α-GalCer and its analogs are widely used as vaccine adjuvants to enhance host immunity to combat infectious diseases (10–14), highlighting the significant roles of iNKT cells in modulating immune responses. In addition to iNKT cell activation, our previous study revealed a unique subset of CD8^+^ T cells with memory-like features were robustly expanded after α-GalCer treatment, termed virtual memory CD8^+^ T (CD8^+^ T_VM_) cells (15).

CD8^+^ T_VM_ cells exhibit memory phenotypes but are present in unimmunized and germ-free mice and even in mice that are fed an elemental diet free of potential food antigens, suggesting that they are antigen-inexperienced (16). Similar to conventional memory CD8^+^ T cells, they expressed high levels of CD44 and CD122 (17). In contrast, CD8^+^ T_VM_ cells express a low level of CD49d, whose expression is only upregulated in true memory CD8^+^ T (CD8^+^ T_MEM_) cells following TCR engagement with cognate antigen (16–19). Moreover, CD8^+^ T_VM_ cells are not only phenotypically similar to conventional memory CD8^+^ T cells but also functionally. CD8^+^ T_VM_ cells have a stronger ability than naïve CD8^+^ T cells (CD8^+^ T_N_) to produce IFN-γ in an antigen-dependent manner (20, 21). Besides, an *in vivo* animal study indicated that ovalbumin (OVA)-specific CD8^+^ T_VM_ cells provided antigen-specific protection against the infection of *Listeria monocytogenes* expressing OVA (21). Interestingly, in addition to antigen-specific activation, CD8^+^ T_VM_ cells can also be activated by cytokines without encountering cognate antigens, a phenomenon called “bystander activation” (22). In *in vitro* settings, CD8^+^ T_VM_ cells can rapidly secrete large amounts of IFN-γ in response to IL-12/IL-18 stimulation compared to naïve T cells (16, 21, 23). *In vivo* studies also showed that CD8^+^ T_VM_ cells exert higher effector functions than naïve T cells in controlling unrelated pathogen infections (22). Similarly, previous reports showed that CD8^+^ T_VM_ cell expansion caused by helminth infection can execute bystander protection against infections by unrelated pathogens (15, 24). Together, these characteristics highlight the uniqueness of CD8^+^ T_VM_ cells as antigen-inexperienced memory CD8^+^ T cells.

Our previous study found that α-GalCer treatment significantly expands the CD8^+^ T_VM_ cell population (15). However, the role of the expanded CD8^+^ T_VM_ cells in α-GalCer treatment-mediated protection against infection remains poorly understood. Given the broad and bystander protection provided by CD8^+^ T_VM_ cells in previous studies, we asked whether, in addition to the iNKT cells, the expanded CD8^+^ T_VM_ cells also play a protective role in α-GalCer treatment-mediated host defense against infections.

Here, we reveal the previously unrecognized roles of expanded CD8^+^ T_VM_ cells in α-GalCer-mediated protection against enteric, systemic, and pulmonary infections. First, we showed that α-GalCer treatment-mediated protection against bacterial and viral infections is positively associated with systemic CD8^+^ T_VM_ cell expansion. Furthermore, we demonstrated the direct protective role of α-GalCer treatment-expanded CD8^+^ T_VM_ cells in these infection models. Mechanistically, we found that IFN-γ- and TNF-α-producing CD8^+^ T cells significantly increase due to the expansion of CD8^+^ T_VM_ cells, which is mainly dependent on IL-4. Finally, we reported that in the absence of IFN-γ signaling, α-GalCer treatment-expanded CD8^+^ T_VM_ cells provide protection against pulmonary influenza A virus infection in a TNF-α-dependent manner. Collectively, our findings link the bystander protective capability of CD8^+^ T_VM_ cells to the α-GalCer/iNKT cell-mediated immunity.

## Materials and methods

### Mice

Wild-type (WT) mice on C57BL/6 (B6) background were purchased from the National Center for Biomodels (NCB) or the Laboratory Animal Center at the National Taiwan University College of Medicine (NTUCM). All of the knockout (KO) mice used in this study were on B6 background. CD1d KO mice were generously provided by Dr. Chyung-Ru Wang (Northwestern University Feinberg School of Medicine) (25). IL-4 KO (KN2) mice were generously provided by Dr. Irah King (McGill University) (26). STAT-1 KO mice were kindly provided by Dr. Chien-Kuo Lee (NTUCM) (27). TNF-α KO and IFN-γ KO mice were obtained by mating TNF-αIFN-γ double KO mice (28) with WT B6 mice at NCB. All KO strains except STAT-1 KO were rederived and bred at NCB. Mice were kept under specific pathogen-free (SPF) conditions at the Laboratory Animal Center at NTUCM. Mice at 6 to 12 weeks of age were used in most experiments, except some experiments included mice up to 20 weeks old. All animal studies were conducted in accordance with the guidelines and approved by the Institutional Animal Care and Use Committee at NTUCM.

### α-GalCer treatment and infections

Mice were administered intravenously with 0.5 µg/mouse of α-GalCer (Avanti Polar Lipids, Inc.) in DPBS (Gibco™) containing 0.1% BSA and < 0.25% DMSO or solvent alone (DPBS containing 0.1% BSA and < 0.25% DMSO). Five or fourteen days later, mice were infected intragastrically with 5 × 10^9^ colony-forming unit (CFU) 10 median lethal dose, MLD of *Yersinia pseudotuberculosis* (*Ypt*, serotype O:1 strain 32777), intraperitoneally with 1 × 10^6^ CFU (5 MLD) of *Listeria monocytogenes* (*Lm*, strain EDG), intranasally with 2.35 × 10^5^ CFU (1 MLD) of *Streptococcus pneumoniae* (*Spn*, ATCC strain 6304), or intranasally with 15 plaque-forming units (PFU) (1 MLD for B6 WT mice, batch #1) or 150 PFU (10 MLD for STAT-1 KO mice, batch #2) of influenza A virus (IAV, H1N1 strain A/PR/8/34). Mice survival and weight were monitored after pathogen infections. Unresponsive or recumbent mice were considered moribund and euthanized. To determine bacterial and viral burdens, the liver, spleen, or lungs were harvested at the indicated times post infection. In some experiments, DMSO- or α-GalCer-treated mice were sacrificed at indicated times, and blood and organs were harvested for further analysis. To inhibit lymphocyte migration from lymphoid organs to lung tissue, mice were intraperitoneally injected with 0.02 mg of FTY720 in 200 μl of ddH2O from the day before a-GalCer treatment until the day before sacrifice. 

### Bacterial burden quantification

Organs were harvested, placed in a 5 ml eppendorf with homogenate beads and 1 ml DPBS, and homogenized by a homogenizer (NEXT ADVANCE). Homogenates were collected and plated onto tryptic soy agar (for *Lm*), Luria-Bertani (LB) agar (for *Ypt*), or blood agar plates (for *Spn*). The plates were then incubated for 1–2 days at 37 °C (for *Lm* and *Spn*) or 26 °C (for *Ypt*). The bacterial colonies were counted, and the CFUs were calculated.

### Lung viral burden quantification

IAV-infected mice were sacrificed after indicated days of infection. Lungs were harvested and immediately frozen in liquid nitrogen. Lungs were then transferred to a 5 ml eppendorf with homogenate beads and 1 ml serum-free infection medium, placed in a homogenizer (NEXT ADVANCE), and homogenized twice at speed 12 for 5 min. The tissue homogenates were frozen at -80 °C for 2 h. Then, lung homogenates were collected and transferred into a 1 ml eppendorf, and centrifuged at 500 × g, 4 °C for 10 min. The supernatant was collected, and the viral titers were determined by plaque assay.

### Flow cytometry

Cells resuspended in 1X DPBS were stained with Zombie Aqua™ Fixable Viability Kit (BioLegend) for 15 min in the dark to discriminate live cells from dead cells. After being washed by FACS buffer (1X DPBS containing 1% FBS and 0.1% NaN_3_), cells were incubated with anti-mouse CD16/32 (BioLegend) to block non-specific bindings. In some experiments, cells were stained with PBS-57-loaded CD1d tetramer (CD1d-α-GalCer tetramer), which was kindly provided by the NIH Tetramer Core Facility, at 4 °C for 1 h in the dark. Cells were then stained with fluorescent-conjugated antibodies against specific surface antigens at 4 °C for 30 min in the dark. Detailed antibody information is provided in **Supplementary Table 1**. Fluorescent-labeled cells were fixed with 1% paraformaldehyde (PFA) and acquired on the full spectrum Cytek^®^ Northern Lights (Cytek Biosciences).

To detect intracellular cytokines, cells harvested from different tissues were seeded in a 96-well plate (0.5–1 × 10^6^ cells/well). Cells were then stimulated with 20 ng/ml of Phorbol myristate acetate (PMA) and 1 µg/ml of ionomycin for 2 h at 37 °C/5% CO_2_ condition. Brefeldin A (5 µg/ml) was added throughout the stimulation. After incubation, cells were stained for surface markers as described above. After surface staining, cells were fixed with 4% PFA at room temperature for 30 minutes, then permeabilized and washed twice with Perm/Wash buffer (eBioscience™). Cells were further stained with fluorochrome-conjugated antibodies against IFN-γ, TNF-α, and granzyme B at 4 °C overnight and acquired on the Cytek^®^ Northern Lights (Cytek Biosciences). Data were analyzed by FlowJo v10 (BD Biosciences) and Prism 8 (GraphPad).

### CD8 depletion

B6 WT mice were injected with α-GalCer or solvent DMSO as described above and then injected intraperitoneally with 0.5 mg of anti-CD8 antibody (clone 2.43, BioXCell), PBS, or Rat IgG2b (clone LTF-2, BioXCell) 13 days later. After one day, mice were infected intragastrically with 5 × 10^9^ CFU of *Ypt*. The spleen and liver were harvested to measure bacterial loads on day 5 after infection.

### Adoptive cell transfer

B6 WT mice or TNF-α KO mice were injected with α-GalCer, and the spleen was harvested 14 days later. Splenic CD8^+^ T cells were enriched by B cell panning using Goat Anti-Mouse IgG + IgM (H+L) (Jackson ImmunoResearch) and further purified by MojoSort™ Mouse CD8 T Cell Isolation Kit (BioLegend) according to the manufacturer’s instructions. CD8^+^ T_N_ (CD44^lo^CD49d^int^) and CD8^+^ T_VM_ (CD44^hi^CD49d^lo^) cells were sorted on BD FACSAria II (BD Biosciences) through the service provided by the Flow Cytometric Analyzing and Sorting Core (the First Core Laboratory, NTUCM). A total of 2.5 × 10^6^ cells of each population were transferred intranasally into STAT-1 KO or intravenously into IFN-γ KO recipients one day before IAV or *Lm* infection, respectively. Lungs were harvested to measure IAV viral titer 4 days after infection. Blood was collected to measure *Lm* CFU 2 days after infection.

### Statistical analysis

Data were plotted and analyzed by Prism 8 (GraphPad software). Statistical differences were analyzed by parametric tests, including unpaired two-tailed Student’s *t* test, one-way ANOVA, and two-way ANOVA, as indicated in the figure legends. The Log-rank test was used to compare survival curves between groups. When the bacterial burden fell below the detection limit of our assays, CFU were assigned values 0.2 log below the detection limit, and consequently, the data were analyzed by nonparametric test (Mann-Whitney). Data are represented as mean ± standard error of the mean (SEM). Experiments were performed at least two times independently, and the specific numbers of experimental replicates and mice were indicated in the respective figure legends. Each symbol represents one individual mouse. A *P* value of <0.05 was considered statistically significant. ns, not significant; *P < 0.05; **P < 0.01; ***P < 0.001; ****P < 0.0001. Experimental procedures were created with BioRender.com.

## Results

### α-GalCer-mediated sustained protection against diverse infections is positively associated with systemic CD8^+^ T_VM_ cell expansion

**Figure 1:**
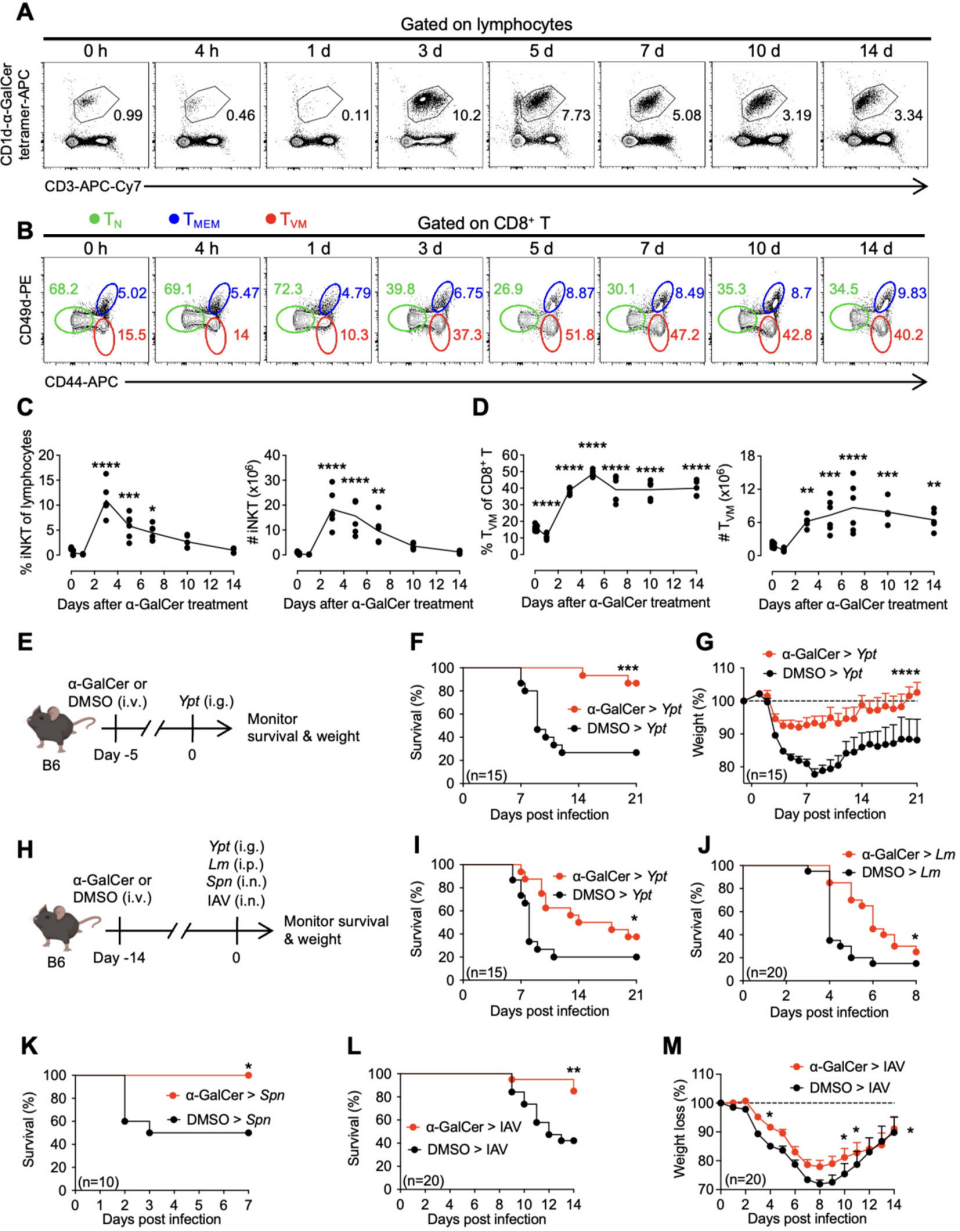
α-GalCer-mediated sustained protection against various infections is positively associated with CD8^+^ T_VM_ cell expansion. **(A–D)** B6 WT mice were injected with α-GalCer, and the spleens were harvested at indicated times. Representative plots of iNKT cells **(A)**, CD8^+^ naïve T (T_N_, labeled in green), true memory (T_MEM_, labeled in blue), and virtual memory (T_VM_, labeled in red) cells **(B)** at indicated times. The percentage and cell numbers of iNKT cells **(C)** and CD8^+^ T_VM_ cells **(D)** in the spleen. **(E–G)** B6 WT mice were treated with α-GalCer or the solvent dimethyl sulfoxide (DMSO) intravenously and infected intragastrically with *Ypt* 5 days later. Survival **(F)** and weight **(G)** were monitored daily after infection. **(H–M)** B6 WT mice were treated with α-GalCer or DMSO intravenously and infected intragastrically with *Ypt* **(I)**, intraperitoneally with *Lm* **(J)**, intranasally with *Spn* **(K)**, or intranasally with Influenza A virus (IAV) **(L, M)** after 14 days. Survival **(I–L)** and weight **(M)** were monitored daily after infections. Data are pooled from two to four independent experiments with a total of five to six **(A–D)** or ten to twenty **(E–M)** mice per group. The line depicts the means **(C, D)**. Data are presented as mean + SEM **(G, M)**. *P < 0.05; **P < 0.01; ***P < 0.001; ****P < 0.0001 by one-way ANOVA as compared to day 0 control **(C, D)**, Log-rank test **(F, I–L)**, or two-way ANOVA **(G, M)**.

To investigate whether CD8^+^ T_VM_ cells are involved in α-GalCer-mediated defense against infections, we first examined the temporal kinetics of iNKT and CD8^+^ T_VM_ cell expansion after α-GalCer treatment. Wild-type (WT) C57BL/6 (B6) mice were injected with α-GalCer intravenously, and spleens were harvested at different time points. Consistent with previous studies (2), the percentages and numbers of iNKT cells (CD3^+^CD1d-α-GalCer tetramer^+^) increased rapidly in the spleens from day 1 to day 3 after α-GalCer treatment, and gradually contracted thereafter (**Figures 1A, C**). Accompanying iNKT cell expansion, there was also a rapid and massive expansion of CD8^+^ T_VM_ cells (CD8^+^CD44^hi^CD49d^lo^) from day 1 to day 5, which was sustained until at least 14 days after α-GalCer treatment (**Figures 1B, D**). To study the differential roles of iNKT cells and CD8^+^ T_VM_ cells in protection against infection, we injected mice with α-GalCer and infected them with a gram-negative bacterium, *Yersinia pseudotuberculosis* (*Ypt*), on day 5 (**Figure 1E**) or day 14 (**Figure 1H**) after α-GalCer treatment. Consistent with published reports (29–31), mice infected with *Ypt* 5 days after α-GalCer treatment exhibited significantly increased survival and reduced weight loss compared to control mice (**Figures 1F, G**). Furthermore, we found that mice infected with *Ypt* 14 days after α-GalCer treatment, a time when iNKT cells diminished, yet CD8^+^ T_VM_ cell numbers were still high, also exhibited significantly improved survival compared to DMSO-treated mice (**Figure 1I**), suggesting that the expanded CD8^+^ T_VM_ cells may contribute to protection at the later time point. Due to the innate-like feature of CD8^+^ T_VM_ cells, we asked whether this protective effect can be extended to other infections. Results showed that mice infected with *Listeria monocytogenes* (*Lm*) peritoneally (**Figure 1J**), *Streptococcus pneumoniae* (*Spn*) intranasally (**Figure 1K**), or influenza A virus (IAV) intranasally (**Figures 1L, M**) 14 days after α-GalCer treatment were protected. Together, these data suggest that the CD8^+^ T_VM_ cells expanded by α-GalCer treatment may play a protective role against diverse infections at a time when iNKT cells are diminished.

**Figure 2:**
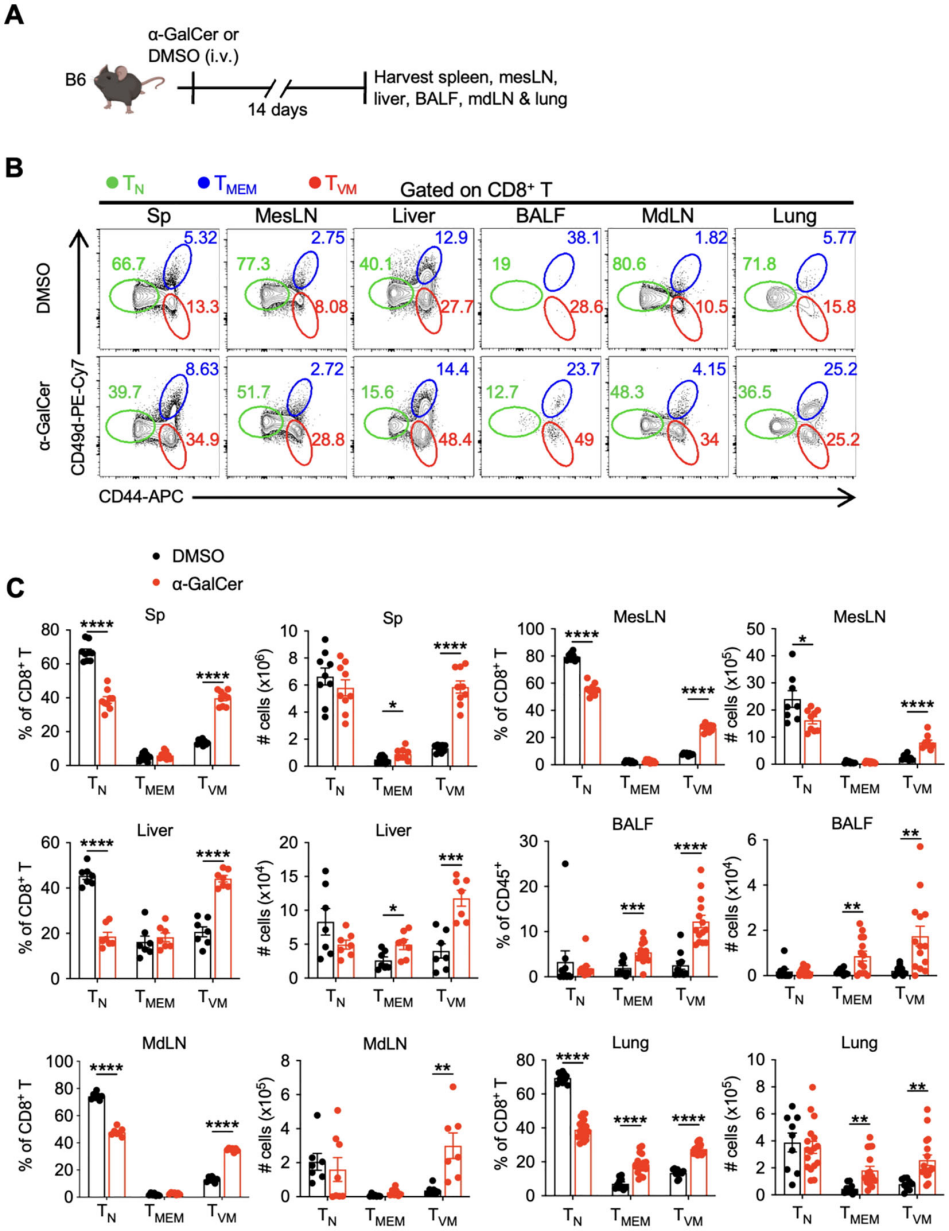
α-GalCer treatment expands CD8^+^ T_VM_ cells systemically. B6 WT mice were injected with α-GalCer, and spleen, MesLN, liver, BALF, MdLN, and lungs after perfusion were harvested and analyzed 14 days later. **(A)** Experimental design. **(B)** Representative flow cytometry plots of CD8^+^ T_N_, T_MEM_, and T_VM_ cells gated on CD8^+^ T cells. **(C)** Percentages and cell numbers of CD8^+^ T_N_, T_MEM_, and T_VM_ cells. Data are pooled from two to four independent experiments and presented as mean ± SEM (n=6–19 per group). *P < 0.05; **P < 0.01; ***P < 0.001, ****P < 0.0001 by Student’s *t* test.

In the meantime, the number of B cells and total CD8^+^ T cells increased, whereas that of NK cells decreased, and cDC1 cells remained unchanged despite a decrease in percentage. (**Supplementary Figure 1**). Interestingly, α-GalCer treatment expanded CD8^+^ T_VM_ cells were found not only in the spleen but also in mesenteric lymph nodes (MesLN), livers, bronchoalveolar lavage fluids (BALF), mediastinal lymph nodes (MdLN), and lungs (**Figure 2**), whereas the number of iNKT cells did not change systemically, except in the lungs (**Supplementary Figure 2**). Together, these findings suggest that α-GalCer treatment expands CD8^+^ T_VM_ cells systemically and in both lymphoid and non-lymphoid tissues, where they are poised to control infections.

### α-GalCer treatment-expanded CD8^+^ T_VM_ cells contribute to protection against various infections

**Figure 3:**
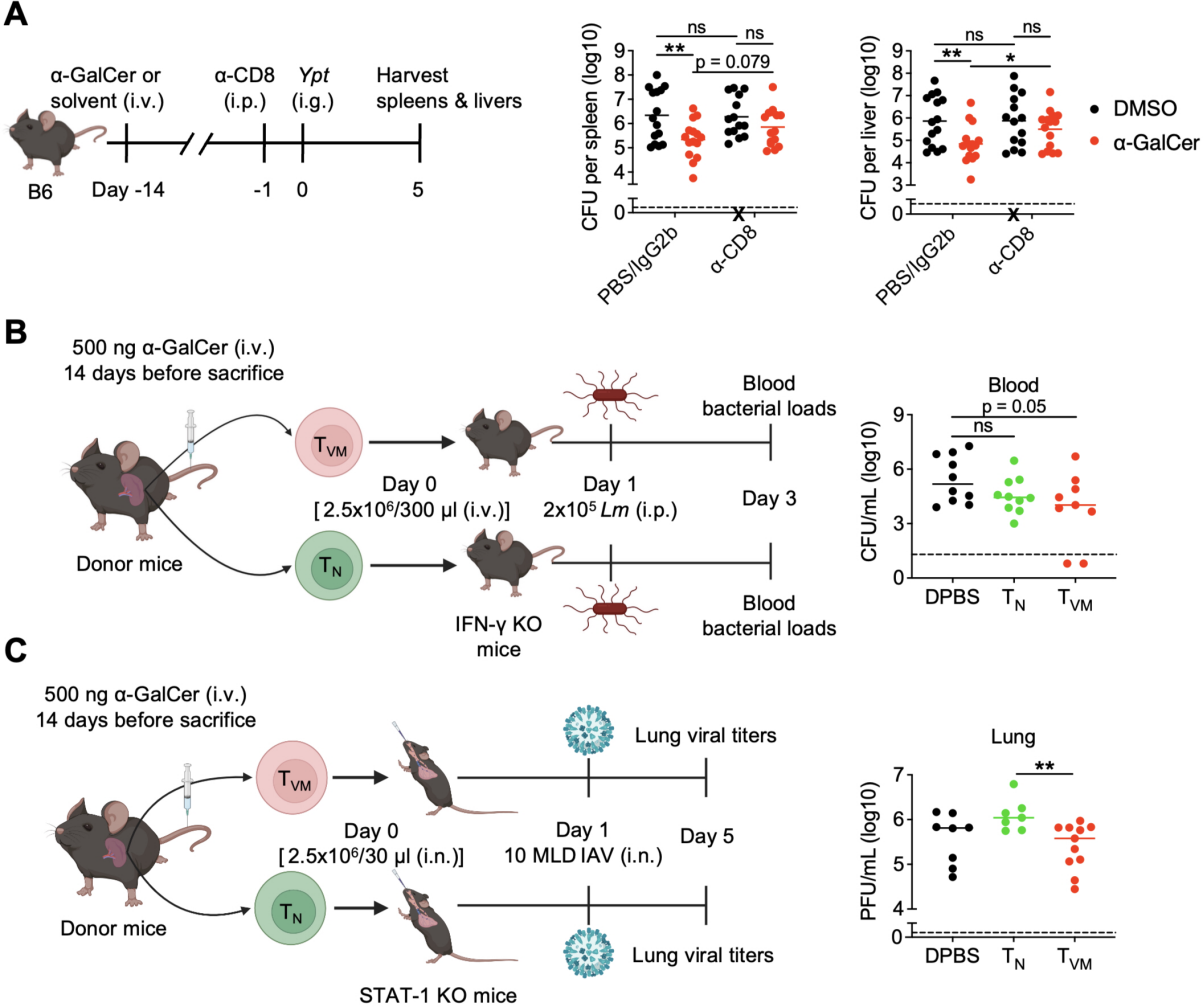
α-GalCer treatment-expanded CD8^+^ T_VM_ cells confer protection against various infections. **(A)** B6 WT mice were injected with α-GalCer or DMSO and then injected intraperitoneally with 0.5 mg of anti-CD8 antibody (α-CD8) or PBS or isotype control (rat IgG2b, LTF-2) 13 days later. After one day, mice were infected intragastrically with *Ypt*. The spleen (left panel) and liver (right panel) were harvested on day 5 after infection, and the bacterial loads were determined (n=15). X indicates a mouse that had succumbed to infection prior to analysis. **(B, C)** B6 WT mice were injected with α-GalCer, and CD8^+^ T_N_ or T_VM_ cells were sorted from the spleen 2 weeks later. A total of 2.5 × 10^6^ cells was transferred into naïve IFN-γ KO **(B)** or STAT-1 KO **(C)** recipients, which were then infected with *Lm* **(B)** or IAV **(C)** the next day, respectively. Bacterial burden in the blood was determined 2 days after infection (n=9-10) **(B)**. Viral titers in the lungs were determined 4 days after infection (n=7–11 per group) **(C)**. The solid bar depicts the mean **(A, C)** or median **(B)**, and the dashed line depicts the limit of detection. Data are pooled from three independent experiments. ns, not significant; *P < 0.05; **P < 0.01 by Student’s *t* test **(A, C)** or non-parametric Mann-Whitney test **(B)**.

To directly link expanded CD8^+^ T_VM_ cells to α-GalCer-induced improved protection, we first performed total CD8^+^ T cell depletion experiments since there is no known method to specifically deplete CD8^+^ T_VM_ cells *in vivo*. While α-GalCer treatment given 14 days before infection reduced *Ypt* burdens in the spleen and liver (**Figure 3A**), depleting CD8^+^ T cells significantly increased bacterial burdens in α-GalCer-treated mice to the levels that were comparable to those of DMSO-treated mice (**Figure 3A**). This result indicated that CD8^+^ T cells play a key role in α-GalCer-mediated protection. Since CD8^+^ T cells in α-GalCer-treated mice were mainly composed of naïve T (T_N_) cells (40-50%) and T_VM_ cells (40-50%) in the spleen (**Figure 2**), we performed adoptive cell transfer experiments to test which population directly contributes to protection after α-GalCer treatment. Splenic CD8^+^ T_N_ and T_VM_ cells from α-GalCer-treated mice were sorted 14 days after treatment, separately transferred to recipient mice, which were then infected with pathogens. Systemic *Lm* infection with intravenous cell transfer and pulmonary IAV infection with intranasal cell transfer models were used for proof-of-concept experiments. To minimize the effects of host endogenous immunity, IFN-γ KO and STAT-1 KO mice were used as recipients for *Lm* and IAV infection, respectively. We found that transfer of α-GalCer treatment-expanded CD8^+^ T_VM_ cells significantly reduced *Lm* loads in the blood (**Figure 3B**) and IAV titers in the lungs (**Figure 3C**). Taken together, these data strongly suggest that expanded CD8^+^ T_VM_ cells play a direct protective role in α-GalCer-mediated host defense against diverse infections.

### α-GalCer treatment-induced CD8^+^ T_VM_ cell expansion contributes to the significant increase of IFN-γ- and TNF-α-producing CD8^+^ T cells

**Figure 4:**
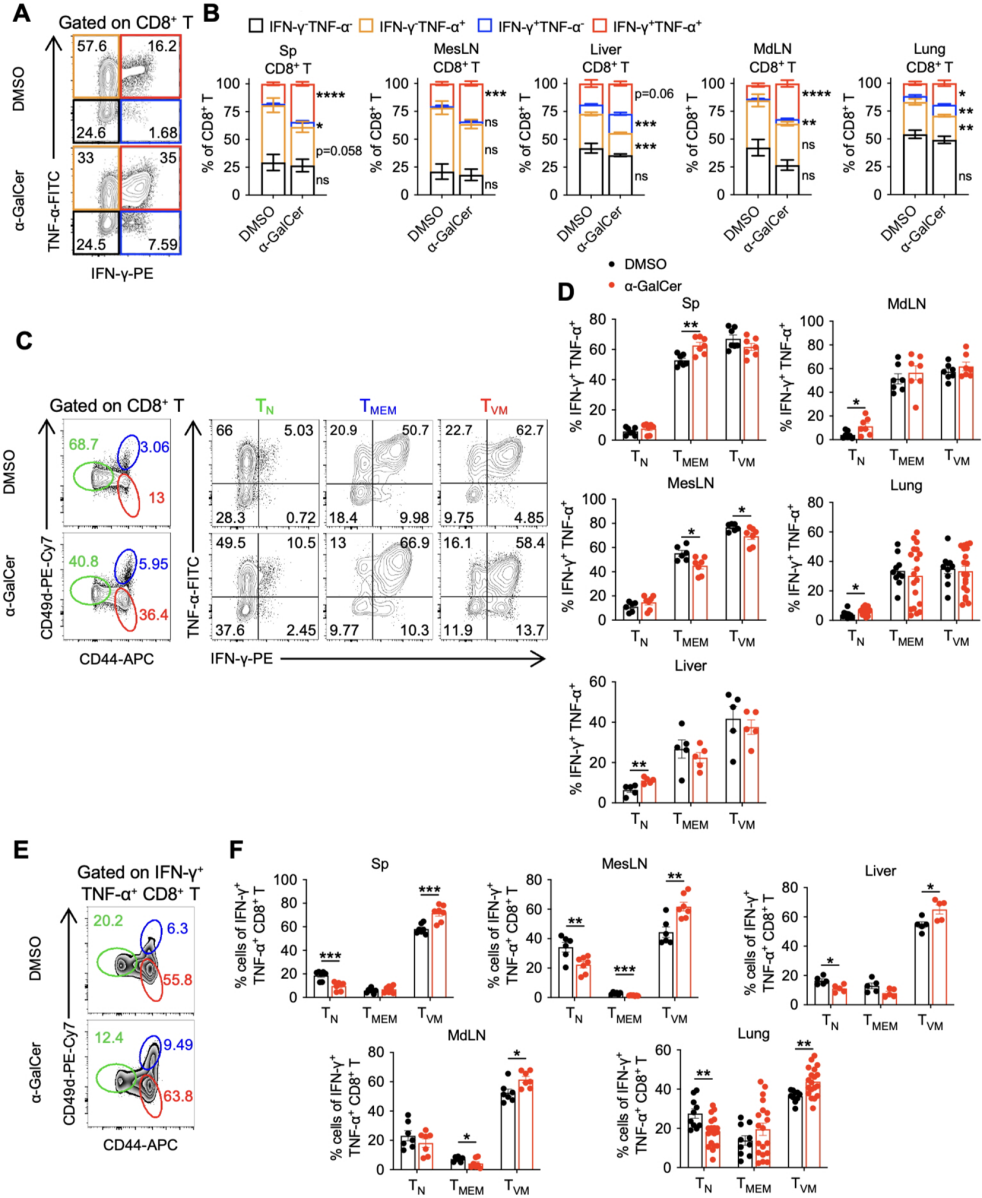
α-GalCer-induced CD8^+^ T_VM_ cell expansion contributes to the significant increase of cytokine-producing CD8^+^ T cells. B6 WT mice were treated with α-GalCer, and indicated tissues were harvested 14 days later. Cells were then stimulated with PMA and ionomycin for 2 h in the presence of brefeldin A. **(A, B)** Representative flow cytometry plots **(A)** and percentages **(B)** of IFN-γ and TNF-α production of CD8^+^ T cells from spleen **(A)** or indicated tissues **(B)**. **(C)** Representative flow cytometry plots of IFN-γ and TNF-α production of splenic CD8^+^ T_N_, T_MEM_, and T_VM_ cells. **(D)** Percentages of IFN-γ^+^TNF-α^+^ CD8^+^ T_N_, T_MEM_, and T_VM_ cells from indicated tissues. **(E, F)** Representative flow cytometry plots **(E)** and percentages **(F)** of CD8^+^ T_N_, T_MEM_, and T_VM_ cells among IFN-γ^+^TNF-α^+^ CD8^+^ T cells from spleen **(E)** or indicated tissues **(F)**. Data are pooled from two to four independent experiments and presented as mean ± SEM (n=5–19 per group). ns, not significant; *P < 0.05; **P < 0.01; ***P < 0.001, ****P < 0.0001 by Student’s *t* test.

Next, we investigated the protective mechanisms of CD8^+^ T_VM_ cells in α-GalCer-mediated sustained protection against infections. Since IFN-γ and TNF-α are key cytokines to control infections (28, 32–34), and CD8^+^ T_VM_ cells are reported to be able to secrete IFN-γ and TNF-α (20, 21), we hypothesized that CD8^+^ T_VM_ cells may contribute to protection by producing IFN-γ and TNF-α. Cells from different tissues of α-GalCer-treated mice were harvested on day 14 after treatment and stimulated with phorbol myristate acetate (PMA) and ionomycin. Intracellular staining revealed that α-GalCer treatment significantly boosted the IFN-γ^+^TNF-α^+^ CD8^+^ T cell populations across various tissues, including the spleen, MesLN, liver, MdLN, and lungs (**Figures 4A, B**). When gated on CD8^+^ T cells and analyzing the ability of T_N_, T_MEM_, and T_VM_ cells to produce IFN-γ and TNF-α, we found that in DMSO-treated control mice, more CD8^+^ T_VM_ cells and T_MEM_ cells than T_N_ cells were capable of producing both cytokines (**Figures 4C, D**), showing their stronger response to PMA/ionomycin *ex vivo* stimulation. Notably, the percentage of IFN-γ^+^TNF-α^+^ CD8^+^ T_VM_ cells was even higher than that of IFN-γ^+^TNF-α^+^ CD8^+^ T_MEM_ cells, suggesting a high cytokine-producing potential of CD8^+^ T_VM_ cells at the steady state. Interestingly, α-GalCer treatment did not further increase the percentage of IFN-γ^+^TNF-α^+^ CD8^+^ T_VM_ cells in these tissues, nor the expression level of each cytokine, as measured by the mean fluorescence intensity (MFI) (**Supplementary Figure 3**). These results suggest that the cytokine-producing potential of each individual CD8^+^ T_VM_ cell is not enhanced by α-GalCer treatment.

We further analyzed the compositions of IFN-γ^+^TNF-α^+^ CD8^+^ T cells. In DMSO-treated control mice, CD8^+^ T_VM_ cells comprised 40-60% of the IFN-γ^+^TNF-α^+^ CD8^+^ T cells (**Figures 4E, F**). Although α-GalCer treatment did not increase the percentage of IFN-γ^+^TNF-α^+^ CD8^+^ T_VM_ cells (**Figures 4C, D**), due to CD8^+^ T_VM_ cell expansion (**Figure 2**), the proportion of CD8^+^ T_VM_ cells in the IFN-γ^+^TNF-α^+^ CD8^+^ T cell population increased compared to that of DMSO-treated control mice (**Figures 4E, F**). In addition to IFN-γ and TNF-α, CD8^+^ T_VM_ cells also expressed granzyme B following *ex vivo* stimulation, but exclusively in the liver regardless of the α-GalCer treatment, suggesting they also have tissue-specific cytotoxic activity (**Supplementary Figure 4**). Collectively, these results suggest that α-GalCer treatment significantly increases the population of IFN-γ- and TNF-α-producing CD8^+^ T cells by proportionally expanding the entire CD8^+^ T_VM_ cell population, including cells capable of producing these two cytokines. The treatment does not enhance the ability of CD8^+^ T_VM_ cells to produce IFN-γ and TNF-α on a per-cell basis.

### IL-4 deficiency limits α-GalCer-mediated CD8^+^ T_VM_ cell expansion, which results in diminished IFN-γ^+^TNF-α^+^ CD8^+^ T cell population and protection

**Figure 5:**
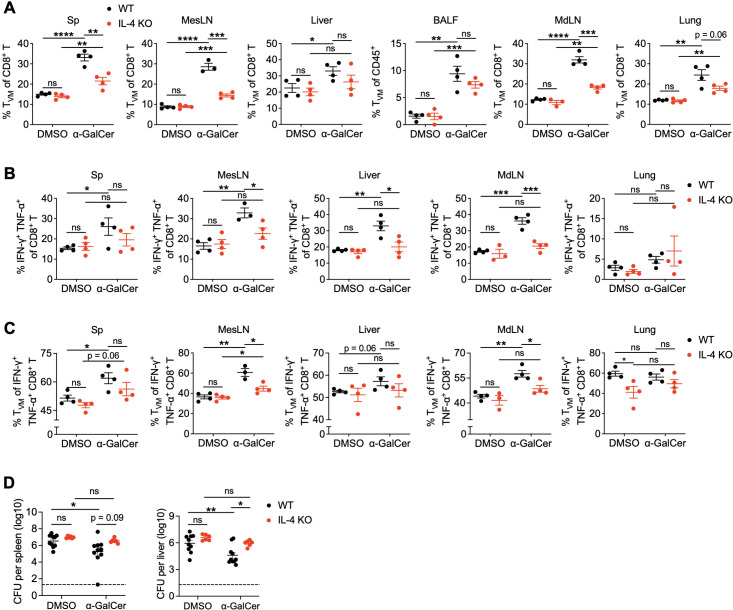
IL-4 deficiency limits the expansion of CD8^+^ T_VM_ cells and abolishes anti-bacterial protection in response to α-GalCer treatment. **(A–C)** B6 WT and IL-4 KO mice were treated with α-GalCer, and indicated tissues were harvested 14 days later. Cells were then stained immediately for flow cytometry analysis or stained after two hours of PMA and ionomycin stimulation in the presence of brefeldin A. **(A)** Percentage of CD8^+^ T_VM_ cells from indicated tissues of B6 WT and IL-4 KO mice. **(B)** Percentages of IFN-γ- and TNF-α-producing CD8^+^ T cells from indicated tissues. **(C)** Percentages of CD8^+^ T_VM_ cells among IFN-γ^+^TNF-α^+^ CD8^+^ T cells from indicated tissues. **(D)** B6 WT and IL-4 KO mice were treated with α-GalCer and infected with *Lm* after 14 days. Bacterial burdens in the spleen (left panel) and liver (right panel) were determined 3 days after infection (n=6-10). The dashed line depicts the limit of detection. Data are pooled from two to three independent experiments with a total of three to four **(A–C)** or six to ten **(D)** mice per group and presented as mean ± SEM. ns, not significant; *P < 0.05; **P < 0.01; ***P < 0.001, ****P < 0.0001 by Student’s *t* test **(A–C)** or one-way ANOVA **(D)**.

Our previous study showed that α-GalCer treatment-induced CD8^+^ T_VM_ cell expansion is mainly IL-4-dependent (15). We found that both the serum level of IL-4 protein (**Supplementary Figure 5A**) and *il4* gene expression in the spleen of WT mice (**Supplementary Figure 5B**) were rapidly upregulated within 4 hours after α-GalCer treatment. Significantly, IL-4 deficiency hampered α-GalCer-induced CD8^+^ T_VM_ cell expansion in the spleen on day 5 (**Supplementary Figure 5C**) and in various tissues on day 14 (**Figure 5A**). It appears that transient expression of IL-4 in the early phase of α-GalCer treatment is sufficient to induce systemic CD8^+^ T_VM_ cell expansion. We next asked whether α-GalCer treatment-induced IFN-γ^+^TNF-α^+^ CD8^+^ T cell expansion is also dependent on IL-4. We found that the percentages of IFN-γ^+^TNF-α^+^ CD8^+^ T cells (**Figure 5B**) as well as the proportion of CD8^+^ T_VM_ cells in the IFN-γ^+^TNF-α^+^ CD8^+^ T cell population (**Figure 5C**) were significantly reduced in the absence of IL-4 compared to IL-4-sufficient mice. These data suggest that IL-4-induced CD8^+^ T_VM_ cell expansion is crucial for optimizing the effector function of total CD8^+^ T cells after α-GalCer treatment.

In addition, consistent with the *in vivo* role of IL-4 in CD8^+^ T_VM_ cell expansion, IL-4 was also required for the maintenance of CD8^+^ T_VM_ cells after α-GalCer stimulation *in vitro* (**Supplementary Figures 6A, B**). To delineate the role of IL-4 in CD8^+^ T_VM_ cell expansion, we asked whether IL-4 supports T_VM_ cell survival or further induces their proliferation. Splenic CD8^+^ T cells were harvested from WT mice, labeled with carboxyfluorescein succinimidyl ester (CFSE), and cultured with IL-4. We found that the presence of IL-4 expanded CD8^+^ T_VM_ cells but not T_N_ cells (**Supplementary Figures 6C–E**) and promoted CD8^+^ T_VM_ cell proliferation (**Supplementary Figures 6F, G**). To directly link α-GalCer-induced IL-4 to the protection against infection *in vivo*, we infected α-GalCer-treated IL-4 KO mice with *Lm* and found that α-GalCer treatment-induced protection against *Lm* was abolished in the absence of IL-4 (**Figure 5D**). Furthermore, we confirmed that α-GalCer treatment-induced T_VM_ cell expansion was completely dependent on the CD1d molecule and iNKT cells (**Supplementary Figure 7**). α-GalCer treatment did not reduce *Lm* burdens in CD1d KO mice (**Supplementary Figure 8**), likely due to iNKT cells being the direct target of α-GalCer (35). Taken together, these results demonstrate the cascade of events after α-GalCer treatment: α-GalCer stimulates iNKT cells to produce IL-4, which expands CD8^+^ T_VM_ cells, and suggest that IL-4 plays an important role in driving CD8^+^ T_VM_ cell proliferation and expansion. CD8^+^ T_VM_ cell proliferation and expansion consequently enrich the IFN-γ^+^TNF-α^+^ CD8^+^ T cell pool to fight against infection.

### Lung and liver CD8^+^ T_VM_ cells expand locally and contribute to the pool of IFN-γ- and TNF-α-producing CD8^+^ T cells

**Figure 6:**
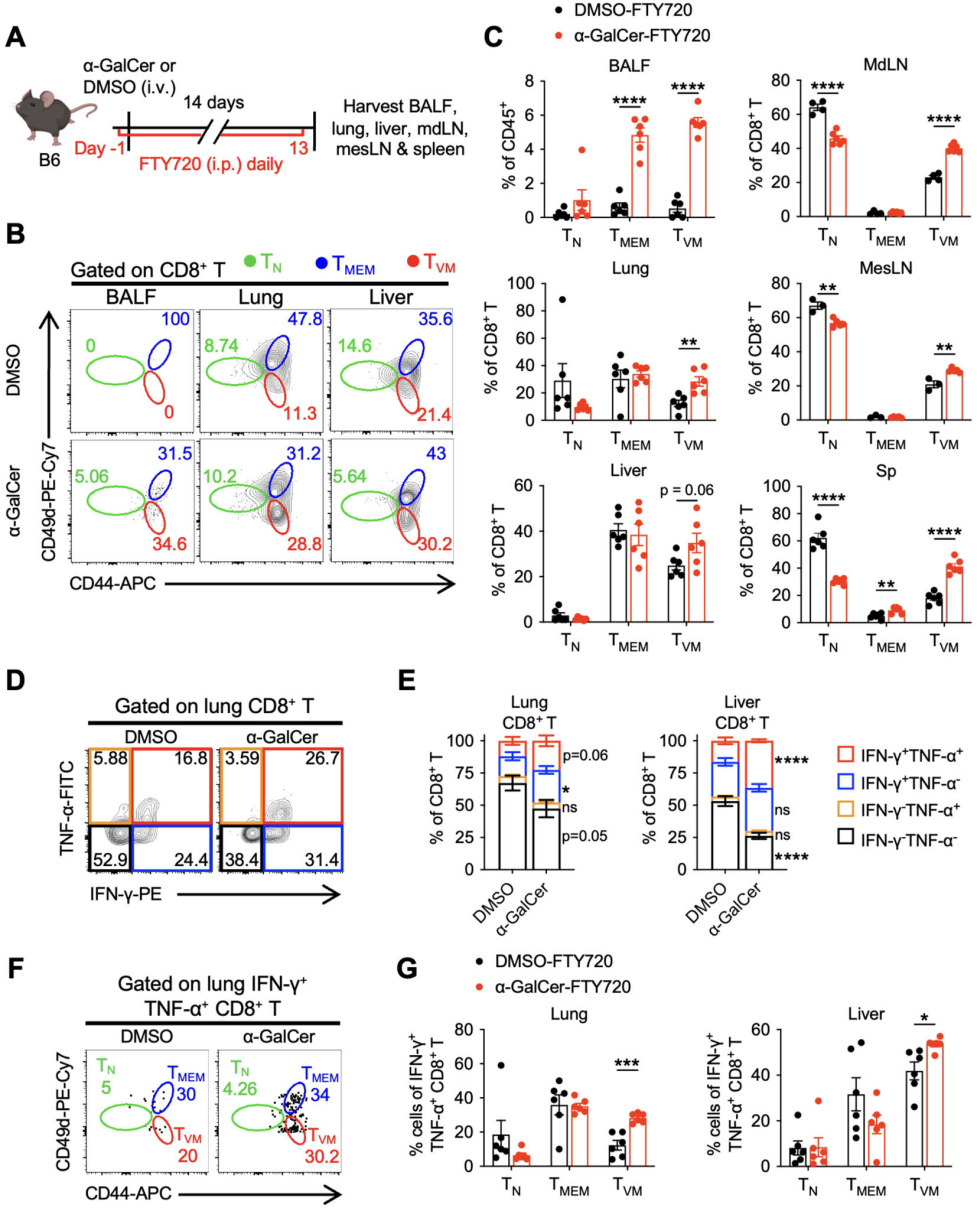
CD8^+^ T_VM_ cells residing in the lung and liver expand locally after α-GalCer treatment. B6 WT mice were intraperitoneally injected with 20 μg of FTY720 one day before α-GalCer treatment. Following daily FTY720 treatment, the cells from indicated tissues were harvested on day 14 after α-GalCer treatment. **(A)** Experimental design. **(B, C)** Representative flow cytometry plots **(B)** and percentages **(C)** of CD8^+^ T_N_, T_MEM_, and T_VM_ cells from indicated tissues. **(D, E)** Representative flow cytometry plots **(D)** and percentages **(E)** of IFN-γ and TNF-α production of lung- and liver-localized CD8^+^ T cells. **(F, G)** Representative flow cytometry plots **(F)** and percentages **(G)** of lung- and liver-localized CD8^+^ T_N_, T_MEM_, and T_VM_ cells among the IFN-γ^+^TNF-α^+^ CD8^+^ T cells. Data are pooled from two experiments and presented as mean ± SEM (n=3–6 per group). *P < 0.05; **P < 0.01; ***P < 0.001; ****P < 0.0001 by Student’s *t* test.

We showed in **Figure 2** that α-GalCer treatment induced CD8^+^ T_VM_ cell expansion in both lymphoid and non-lymphoid tissues, enabling them to rapidly respond to infection locally. Here, we asked whether lung- and liver-localized CD8^+^ T_VM_ cells that reside locally at the steady state are the origin of α-GalCer-expanded CD8^+^ T_VM_ cells and become part of the IFN-γ^+^TNF-α^+^ CD8^+^ T cell pool. Mice were given FTY720 intraperitoneally to block circulating CD8^+^ T_VM_ cells from entering the lung and liver one day before and daily after α-GalCer treatment, then harvested on day 14 after α-GalCer treatment (**Figure 6A**). Consistent with what was reported (36), FTY720 treatment dramatically reduced CD8^+^ T_N_ cells in the lung and liver of DMSO-treated mice (**Figure 6B**), as compared to mice that were not treated with FTY720 (**Figure 2**). In contrast, in mice treated with α-GalCer, FTY720 administration did not abolish the expansion of CD8^+^ T_VM_ cells in BALF, lung, and liver (**Figures 6B, C**), suggesting that CD8^+^ T_VM_ cells present in the lung and liver at steady state can expand locally after α-GalCer treatment. Interestingly, FTY720 treatment did not impede CD8^+^ T_VM_ cell expansion in the lymphoid tissues, such as MdLN, MesLN, and spleen (**Figure 6C**). Since FTY720 treatment does not block the homing of CD8^+^ T cells to lymphoid tissues (37), it remains to be determined whether expanded CD8^+^ T_VM_ cells in these lymphoid tissues were ingressed from non-lymphoid tissues.

Next, tissue-localized CD8^+^ T cells from FTY720-treated mice were harvested and stimulated with PMA and ionomycin *ex vivo* to assess their ability to produce cytokines. The percentages of IFN-γ^+^TNF-α^+^ CD8^+^ T cells increased significantly in the liver and slightly in the lungs after α-GalCer and FTY720 treatment (**Figures 6D, E**). Moreover, lung and liver locally expanded CD8^+^ T_VM_ cells constituted a major population of TNF-α- and IFN-γ-producing CD8^+^ T cells after α-GalCer treatment (**Figures 6F, G**). Together, these results demonstrate that CD8^+^ T_VM_ cells that are present in the lung and liver at the steady state, either as residents or as passersby through circulation, can expand locally in response to α-GalCer treatment and constitute a major population of the TNF-α- and IFN-γ-producing CD8^+^ T cell pool.

### α-GalCer treatment-expanded CD8^+^ T_VM_ cells confer protection against IAV infection in a TNF-α-dependent manner in the absence of IFN-γ signaling

**Figure 7:**
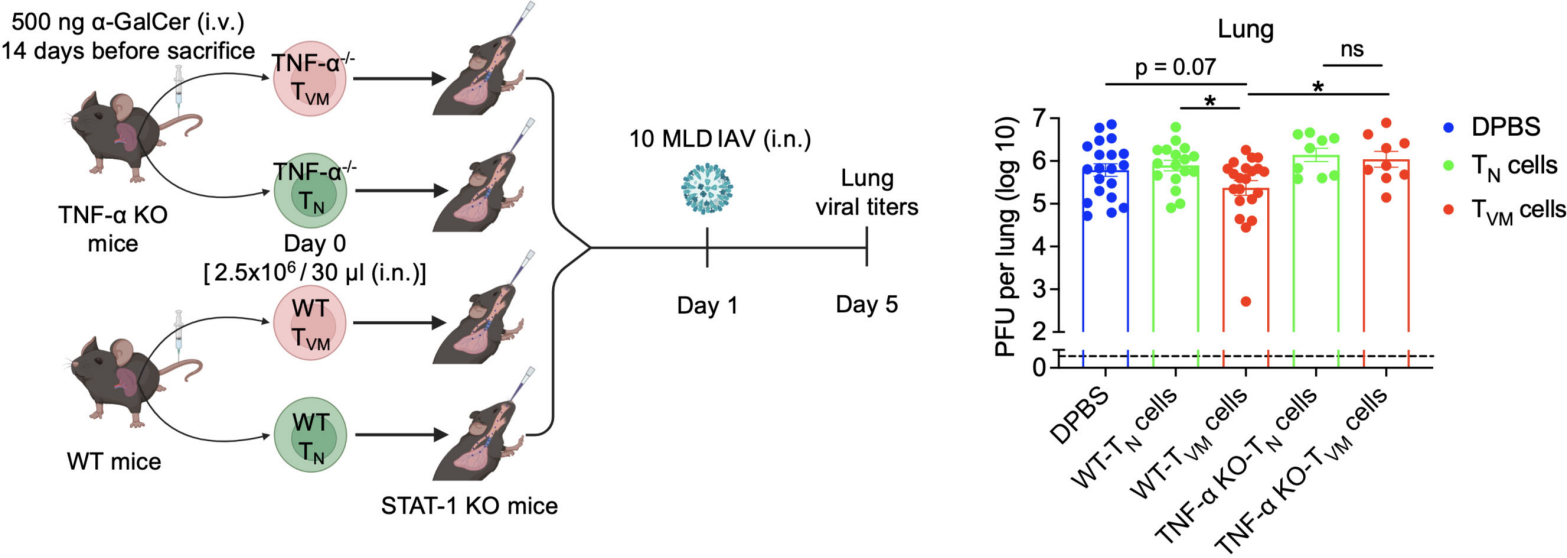
α-GalCer treatment-expanded CD8^+^ T_VM_ cells control IAV infection in a TNF-α-dependent manner. B6 WT mice or TNF-α KO mice were treated with α-GalCer for 14 days. Spleens were harvested, and the CD8^+^ T_N_ and T_VM_ cells were sorted out. A total of two and a half million cells were intranasally transferred to the STAT-1 KO recipient mice, which were then infected with 10 MLD IAV the next day. Lung viral titers were determined 4 days after infection. Data are pooled from four to seven independent experiments with nine to 21 mice per group, including data in **Figure 3C**. Data are presented as mean ± SEM. ns, not significant; *P < 0.05 by Student’s *t* test.

Given that the α-GalCer-expanded CD8^+^ T_VM_ cells conferred protection by reducing viral loads in the lung of IAV-infected mice (**Figure 3C**), and they have a strong ability to secrete TNF-α and IFN-γ (**Figures 4**–**6**), we next investigated CD8^+^ T_VM_ cell-mediated protective mechanisms. In our adoptive transfer-IAV infection model, the STAT-1 KO recipient mice failed to mount transcriptional responses to not only IFN-α but also IFN-γ (38). Due to impaired IFN-γ signaling in STAT-1 KO recipients, transferred WT CD8^+^ T_VM_ cells could not mediate protection through IFN-γ signaling, even though they may be producing IFN-γ during IAV infection. This suggests another factor may be in play. Since large numbers of α-GalCer-expanded CD8^+^ T_VM_ cells produce TNF-α and/or IFN-γ, we hypothesized that TNF-α may play a role in conferring protection against IAV in the absence of IFN-γ. To test this hypothesis, we sorted CD8^+^ T_VM_ cells from WT or TNF-α KO mice and transferred them to STAT-1 KO recipients (**Figure 7**). Consistent with the results shown in **Figure 3C**, mice receiving WT CD8^+^ T_VM_ cells exhibited significantly reduced lung viral titers compared to those receiving WT CD8^+^ T_N_ cells (**Figure 7**). In contrast, mice receiving TNF-α KO CD8^+^ T_VM_ cells showed elevated viral titers in the lungs compared to mice receiving WT CD8^+^ T_VM_ cells, and the levels were comparable to mice receiving WT CD8^+^ T_N_ or TNF-α KO CD8^+^ T_N_ cells (**Figure 7**). It is apparent that in the absence of TNF-α, the transferred CD8^+^ T_VM_ cells lost their ability to defend against IAV infection in the STAT-1 KO mice. These data indicate that in the absence of IFN-γ signaling, α-GalCer-treatment-expanded CD8^+^ T_VM_ cells control IAV infection through a TNF-α-dependent mechanism.

## Discussion

α-GalCer treatment has been widely used to combat various infections and tumors through the activation of innate-like iNKT cells (39–41). In tumor models, iNKT cell-derived IFN-γ enhances NK cell-mediated elimination of MHC class I-negative tumors and promotes CD8^+^ T cell cytotoxicity against MHC class I-positive tumors (8). Transfer of α-GalCer-pulsed DCs activates iNKT cells to promote potent anti-tumor immunity (42–44). In infections, α-GalCer treatment enhances macrophage killing of *Lm* (29) and confers early host defense against *Spn* by enhancing neutrophil recruitment (31, 45). Moreover, co-administration of α-GalCer with influenza vaccines or inactivated virus provides protection against lethal influenza challenge (46–48). However, these studies primarily focused on early immune enhancement by co-administering α-GalCer with pathogens or tumors to assess immediate protection (11, 12, 29–31, 45, 46, 49). Whether α-GalCer administration provides long-term beneficial effects has rarely been investigated, and the contribution of expanded CD8^+^ T_VM_ cells is neglected. We discovered in this study that α-GalCer treatment provided protection against various infections not only 5 days but also 14 days after treatment, a time point when iNKT cells had diminished, but CD8^+^ T_VM_ cells remained systemically expanded. CD8 depletion and adoptive transfer experiments demonstrated that CD8^+^ T_VM_ cells directly contribute to α-GalCer-mediated protection. Our findings establish a cascade of immune events in α-GalCer-induced responses, highlight the previously unrecognized contribution of expanded CD8^+^ T_VM_ cells, distinct from traditionally emphasized iNKT cells, in α-GalCer treatment-mediated protection, and provide important insights into the unique role of CD8^+^ T_VM_ cells in bridging innate and adaptive immunity.

Our findings also shed light on the mechanisms by which CD8^+^ T_VM_ cells protect against various infections. Functionally, CD8^+^ T_VM_ cells exhibited strong effector potential. Previous studies have shown that CD8^+^ T_VM_ cells rapidly produce IFN-γ or TNF-α in response to IL-12 and IL-18 culture as well as cognate antigen stimulation, which is superior to T_N_ cells but less competent than T_MEM_ cells (16, 20, 21). Here, we found that upon *ex vivo* PMA and ionomycin stimulation, CD8^+^ T_VM_ cells produced more IFN-γ^+^ and TNF-α^+^ than T_N_ cells and were comparable to T_MEM_ cells. In some organs, there were even higher percentages of IFN-γ^+^TNF-α^+^ CD8^+^ T_VM_ cells than IFN-γ^+^TNF-α^+^ T_MEM_ cells. Collectively, these findings reflect that the activation threshold is much lower in CD8^+^ T_VM_ cells than in CD8^+^ T_N_ cells. Interestingly, we found that α-GalCer treatment did not directly boost the capacity of CD8^+^ T_VM_ cells to produce IFN-γ and TNF-α on a per-cell basis, but instead expanded CD8^+^ T_VM_ cell numbers to contribute to the IFN-γ^+^TNF-α^+^ CD8^+^ T cell pool.

In addition to IFN-γ and TNF-α, we observed that liver CD8^+^ T_VM_ cells exclusively expressed granzyme B upon *ex vivo* stimulation. One possible explanation is that hepatic stellate cells, uniquely present in the liver, help maintain the homeostasis of memory CD8^+^ T cells via IL-15 transpresentation (50). Previous studies have highlighted the critical role of IL-15 in granzyme B secretion by CD8^+^ T_VM_ cells (51). It is likely that IL-15 from hepatic stellate cells signals CD8^+^ T_VM_ cells in the liver, enabling them to secrete granzyme B. These findings hint that tissue-specific niche influences the effector functions of CD8^+^ T_VM_ cells, supporting the notion that functional heterogeneity exists for CD8^+^ T_VM_ cells, and it is anatomically diverse.

TNF-α has been recognized as a double-edged sword during IAV infection. It was previously reported that TNF-α can inhibit the replication of IAV in lung epithelial cells *in vitro* (52) and enhance the expression of IL-28 and IL-29 in human lung epithelial cells (34). In contrast, other studies have shown that inhibition of TNF-α reduces IAV-induced lung pathology, accompanied by a reduction of immune cell infiltration (53). Despite the controversial roles of TNF-α in IAV infection, little is known about the specific cellular source of TNF-α that mediates these effects.

Here, we observed that α-GalCer-expanded CD8^+^ T_VM_ cells control infectious IAV in STAT-1 KO mice through a TNF-α-dependent mechanism. The result provides strong *in vivo* evidence that TNF-α exerts a potent anti-IAV effect to lower viral titers, particularly in the absence of IFN-γ. However, we did not examine the long-term survival or lung pathology in mice receiving WT or TNF-α-deficient CD8^+^ T_VM_ cells after IAV challenge. Therefore, whether TNF-α produced by α-GalCer-expanded CD8^+^ T_VM_ cells is sufficient to confer an overall beneficial effect during IAV infection remains to be investigated. We cannot rule out the potential contribution of IFN-γ in CD8^+^ T_VM_ cell-mediated anti-IAV immunity, either. Nevertheless, our findings provide a new perspective on CD8^+^ T_VM_ cells as a critical source of TNF-α, thereby contributing to anti-IAV immunity.

The result of our study confirmed that the expansion of CD8^+^ T_VM_ cells induced by α-GalCer treatment is predominantly dependent on IL-4, which requires prior activation of iNKT cells. In line with this result, IL-4 is also critical for α-GalCer-mediated protection against infection. Contradictorily, IL-4 is considered a signature T_H_2 cytokine that antagonizes T_H_1 and CD8^+^ T cell responses (54). There are studies showing that IL-4 induces the expansion of IFN-γ-expressing CD44^hi^CXCR3^+^CD8^+^ cells (55), and short-term IL-4 exposure during the development or priming stage induces durable CD8^+^ T cell memory responses (56, 57). In this study, we observed that IL-4 was transiently induced after α-GalCer treatment but declined shortly thereafter, which was followed by a robust expansion of CD8^+^ T_VM_ cells, resulting in stronger CD8^+^ T immunity. Altogether, these findings support the concept that transient IL-4 signaling may contribute to the generation of functional CD8^+^ T cell memory responses. A previous report revealed that α-GalCer-activated iNKT cells promote memory CD8^+^ T cell (CD44^hi^CD62L^hi^) differentiation during murine cytomegalovirus infection (49). Since CD8^+^ T_VM_ cells share the same phenotypes (CD44^hi^CXCR3^+^CD62L^hi^) with CD44^hi^CXCR3^+^CD8^+^ cells and the memory CD8^+^ cells (CD44^hi^CD62L^hi^), it is conceivable that CD8^+^ T_VM_ cells are the cells that respond to IL-4 in previous reports.

While IL-4 is important in driving CD8^+^ T_VM_ cell proliferation and expansion, there seems to be an IL-4-independent pathway that may involve other cytokines. Our preliminary results showed that in addition to IL-4, serum IFN-α1 and splenic gene expressions of *il2*, *il15*, *il13*, *ifng*, *il6*, *il1b*, and *il17a* were also upregulated upon α-GalCer treatment. Since IL-15 and type I IFNs have been reported to be involved in CD8^+^ T_VM_ cell development and expansion (51, 58), and IL-2 is important for T cell growth (59), we speculated that any of IL-15, IFN-α1, and IL-2 may be involved in IL-4-independent CD8^+^ T_VM_ cell expansion. It will require further studies to clarify the contribution of these cytokines to CD8^+^ T_VM_ cell expansion in response to α-GalCer treatment.

Dissociating CD8^+^ T_VM_ cells from iNKT cells after α-GalCer treatment is challenging because CD8^+^ T_VM_ cells require IL-4 produced by iNKT cells to expand. For the gain-of-function study, we used an adoptive cell transfer strategy to demonstrate that α-GalCer treatment-expanded CD8^+^ T_VM_ cells are sufficient to confer protection against multiple infections. For the *Lm* model, we used IFN-γ KO mice as recipients to avoid the possibility that IFN-γ produced by host cells masks the potential protective effect of the transferred CD8^+^ T_VM_ cells (32). For the IAV model, we used STAT-1 KO mice to minimize the effects of innate IFN-α and IFN-γ (38). We reasoned that IFN-γ KO and STAT-1 KO mice are relatively more susceptible to *Lm* and IAV infection, respectively, than WT mice. Thus, using these two strains of mice as recipients may provide a better chance to observe protection conferred by CD8^+^ T_VM_ cells, whose protective function may not be as strong as Ag-specific T cells. In fact, we have tried using WT mice as recipients, but the protective effect of transferred CD8^+^ T_VM_ cells in the WT recipients was mild and did not reach statistical significance. We are aware that this approach may not precisely mimic the physiological condition after α-GalCer treatment due to several factors, including the number of transferred cells, the available space in the recipient for the transferred cells, cell recovery and distribution in tissues, and host endogenous immunity. While we demonstrated that α-GalCer-expanded CD8^+^ T_VM_ cells can provide protection against infection, we did not exclude the possibility that other cells may also contribute to α-GalCer-mediated protection. Whether CD8^+^ T_VM_ cells are absolutely required for α-GalCer-mediated protection at later times under physiological conditions warrants further investigation.

For the loss-of-function study, we used a CD8^+^ T cell depletion strategy to demonstrate that CD8^+^ T cells contribute to protection. To the best of our knowledge, there is no known method to specifically delete or knock out CD8^+^ T_VM_ cells, largely due to the lack of specific and reliable markers, molecules, or transcription factors that are unique to CD8^+^ T_VM_ cells. Markers that are highly expressed on CD8^+^ T_VM_ cells, such as Eomesodermin (Eomes), CD44, and CD122, are also expressed on other cells (19, 60). While IL-15 KO and Eomes KO mice show a dramatic reduction of CD8^+^ T_VM_ cell numbers (51, 60, 61), both IL-15 and Eomes are also required for NK cell and effector T cell development and function (62, 63). Therefore, a CD8^+^ T_VM_ cell-specific knockout mouse model has yet to be developed. Regardless of these limitations, the robustness of our study designs provides solid support for the conclusion that α-GalCer treatment-expanded CD8^+^ T_VM_ cells are sufficient to confer protection against a diverse range of infections. Given the broad application of α-GalCer in host defense against pathogens, in anti-tumor therapies, and as vaccine adjuvants, these “memory-like innate” CD8^+^ T_VM_ cells may inevitably play critical roles in the beneficial outcome of α-GalCer-induced NKT cell-mediated immunotherapeutic effects, not only systemically but also at the mucosal sites.

## Data Availability

The raw data supporting the conclusions of this article will be made available by the authors, without undue reservation.
