# Supplementary material for: α-Galactosylceramide-expanded virtual memory CD8+ T cells confer protection against a broad range of pathogens
Source: Front Immunol. 2026 May 22;17:1799271. doi: 10.3389/fimmu.2026.1799271 (PMC13236905; doi:10.3389/fimmu.2026.1799271)
Supplement: Supplementary file 1 [file DataSheet1.pdf]

Supplementary Materials for

α-Galactosylceramide-expanded virtual memory $CD8^+$ T cells confer protection against a broad range of pathogens

Jia-Xun Xie *et al*.

*Corresponding author: Jr-Shiuan Lin, jrshiuanlin@ntu.edu.tw

The file includes:

Materials and Methods

Figures S1 to S8

Tables S1 to S2

Supplementary Materials and Methods

Ex vivo α-GalCer treatment

Single cell suspensions prepared from spleens of naïve B6 WT mice were seeded in a 48-well plate (1.5×10^6 cells/well) and cultured in complete medium containing DMSO (control) or 100 ng/ml of α-GalCer in the presence or absence of 10 µg/ml of anti-mouse IL-4 (clone 11B11) or isotype Rat IgG1 (clone HRPN) antibodies for 3 days. The antibodies were purchased from BioXcell and are listed in Supplementary Table S1. Cells were harvested and processed for further FACS analysis.

$CD8^+$ T cell culture and CFSE labeling

Splenic $CD8^+$ T cells were enriched by B cell panning using Goat Anti-Mouse IgG + IgM (H+L) (Jackson ImmunoResearch) and further purified by MojoSort™ Mouse CD8 T Cell Isolation Kit (BioLegend) according to the manufacturer's instructions. Purified $CD8^+$ T cells were labeled with 0.1 µM CFSE (Invitrogen) for monitoring cell proliferation. CFSE-labeled $CD8^+$ T cells were seeded in a 96-well plate (2.5×10^5 cells/well) and cultured in complete medium or supplemented with 20 ng/ml of recombinant murine IL-4 (PeproTech) for 3 days at 37°C, 5% CO_2 condition. After incubation, cells were harvested and processed for FACS analysis.

Quantitative PCR (qPCR)

Total RNA was extracted by Direct-zol RNA Miniprep Kit (ZYMO RESEARCH) according to the manufacturer's instructions. RNA purity was measured by DS-11 Spectrophotometer (DeNovix). Following the ToolsQuant II Fast RT Kit (BIOTOOLS) instructions, 1000 ng of RNA was reverse transcribed to complementary DNA (cDNA). Quantitative PCR (qPCR) was carried out using the qPCRBIO SyGreen Mix Lo-ROX kit (PCR Biosystems) according to the manufacturer's instructions. Briefly, a total of 25-50 ng of cDNA was amplified by the QuantStudio™ 3 Real-Time PCR System (Applied Biosystems™) with a total volume of 20 µl per reaction. mRNA expression level of the target gene was normalized to that of *gapdh* and expressed as relative expression ($2^{-\Delta Ct}$). The fold change ($2^{-\Delta\Delta Ct}$) reflected the relative expression of the treatment group to the control group. Primer sequences are provided in Supplementary Table S2.

Enzyme-linked immunosorbent assay (ELISA)

To measure cytokines in the serum, ELISA MAX™ Standard Set Mouse IL-4 and IFN-α1 kits (BioLegend) were used according to the manufacturer's instructions. OD450 absorbance was detected by iMark™ Microplate Absorbance Reader (Bio-Rad).

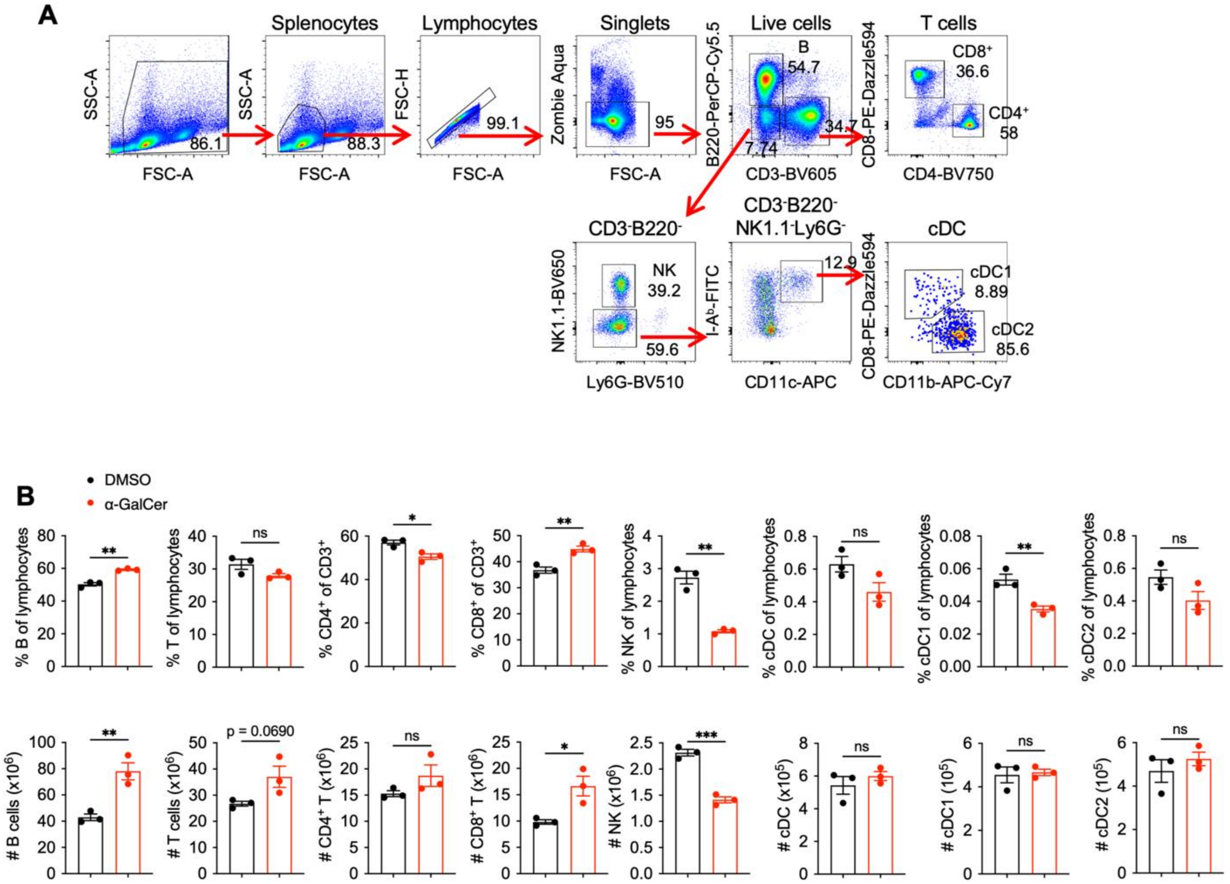

FIGURE S1

Immune cell compositions in the spleen on day 14 after α-GalCer treatment. B6 WT mice were injected with α-GalCer or DMSO, and the spleens were harvested and analyzed 14 days later. **(A)** Gating strategy. **(B)** Percentage (upper) and absolute cell numbers (lower) of immune cells in the spleen. Data are presented as mean ± SEM (n=3). ns, not significant; $*P < 0.05$; $**P < 0.01$; $***P < 0.001$; $****P < 0.0001$ by Student's *t* test.

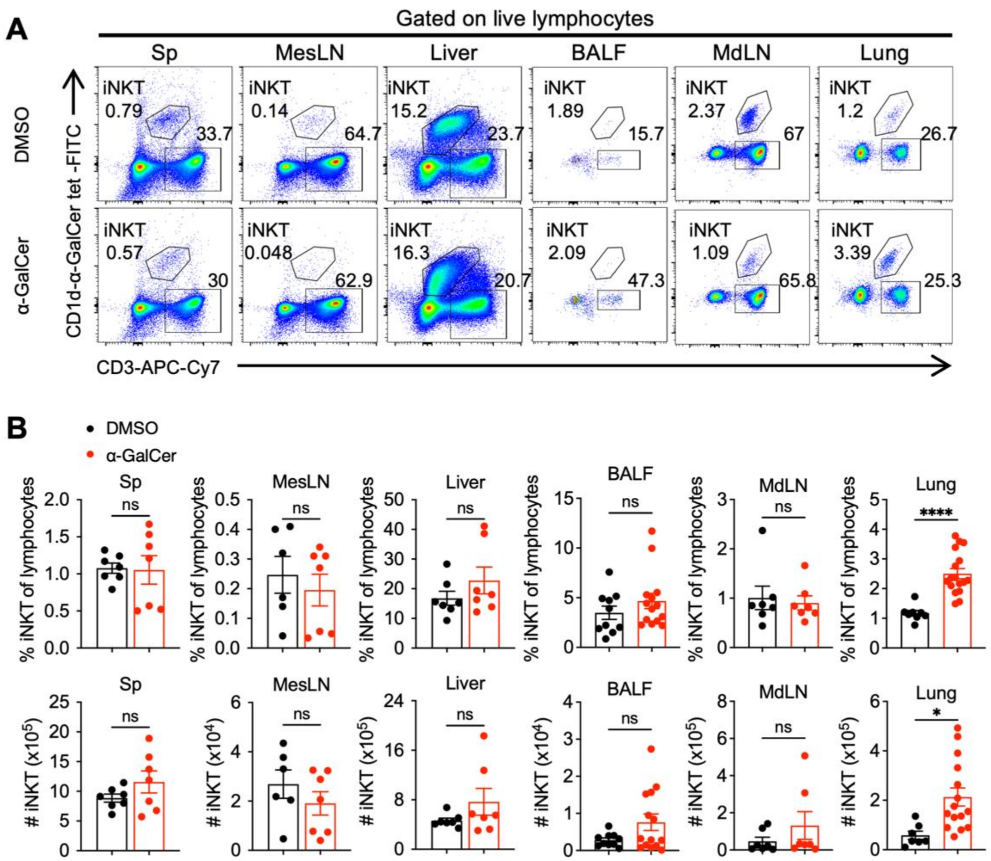

FIGURE S2

iNKT cell populations in different tissues after α-GalCer treatment. B6 WT mice were injected with α-GalCer, and indicated tissues were harvested and analyzed 14 days later. **(A)** Representative flow cytometry plots of iNKT cells. **(B)** Percentage (upper) and absolute cell numbers (lower) of iNKT cells. Data are pooled from two to four independent experiments with a total of six to 19 mice per group. Data are presented as mean ± SEM. ns, not significant; *P < 0.05; **P < 0.01; ***P < 0.001; ****P < 0.0001 by Student's t test.

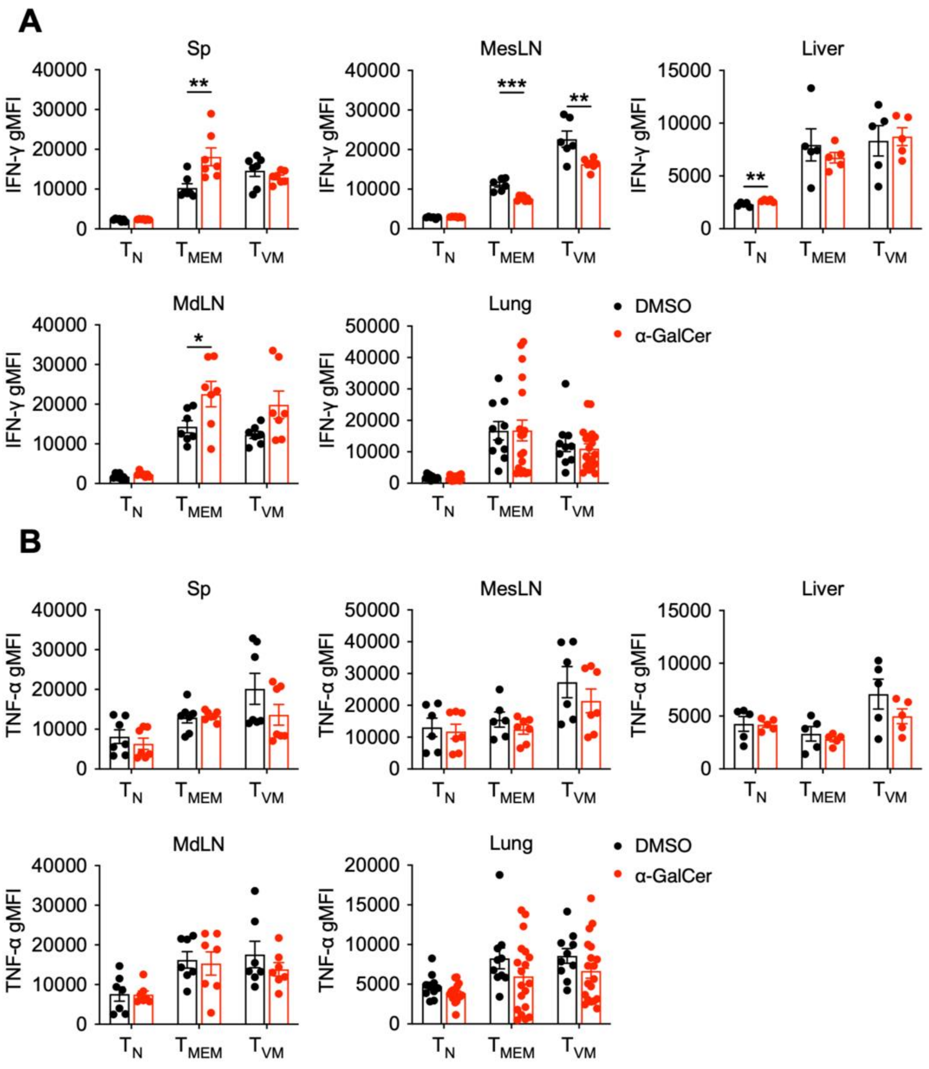

FIGURE S3

The production of IFN-γ and TNF-α by CD8$^+$ T_{VM} cells remains unchanged after α-GalCer treatment. B6 WT mice were injected with α-GalCer, and the indicated tissues were harvested and analyzed 14 days later. **(A, B)** The geometric mean fluorescence intensity (gMFI) of IFN-γ **(A)** and TNF-α **(B)** produced by CD8$^+$ T_N, T_{MEM}, and T_{VM} cells from the indicated tissues. Data are pooled from two to four independent experiments and presented as mean ± SEM (n=5-19 per group). ns, not significant; *$P < 0.05$; **$P < 0.01$; ***$P < 0.001$ by Student's *t* test.

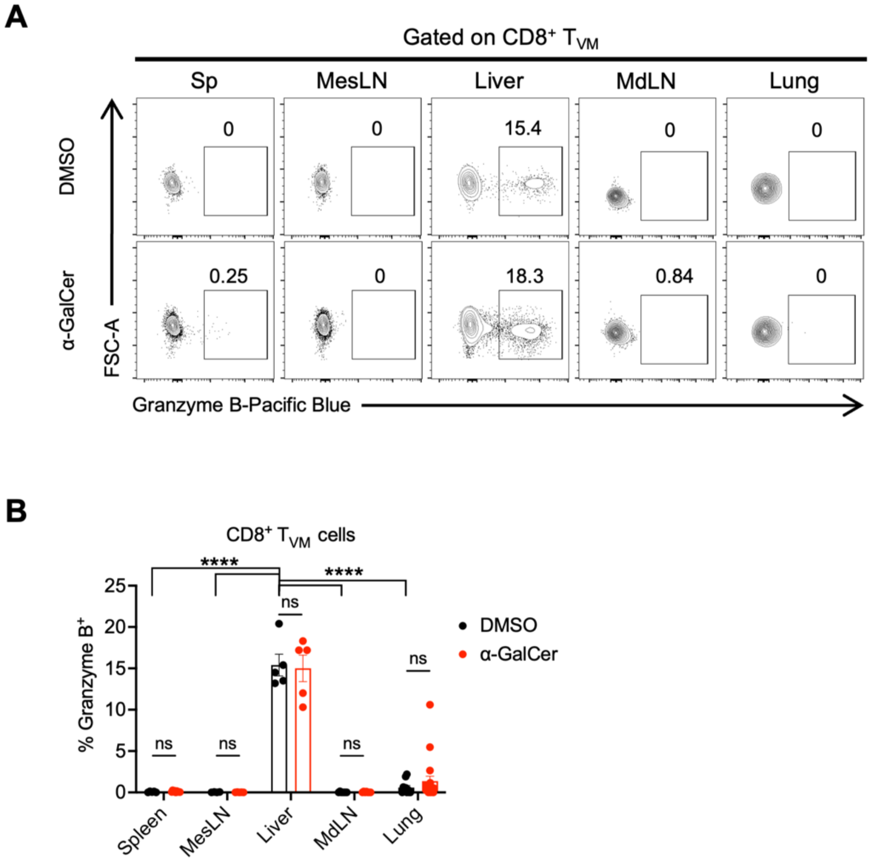

FIGURE S4

Granzyme B expression in CD8$^+$ T$_{VM}$ cells is specifically increased in the liver. B6 WT mice were injected with α-GalCer, and the indicated tissues were harvested and analyzed 14 days later. **(A, B)** Representative flow cytometry plots **(A)** and percentages **(B)** of granzyme B production of CD8$^+$ T$_{VM}$ cells from the indicated tissues. Data are pooled from two to four independent experiments and presented as mean ± SEM (n=5-19 per group). ns, not significant; ****P < 0.0001 by Student's *t* test.

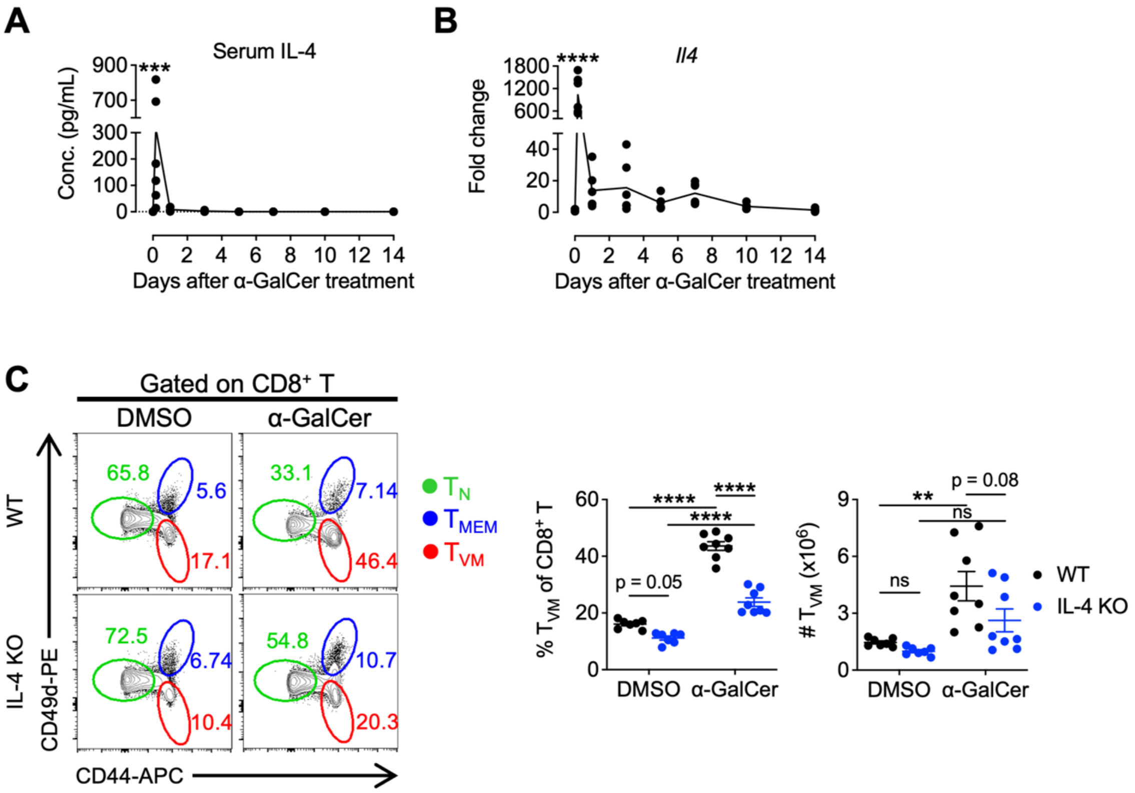

FIGURE S5

α-GalCer-induced CD8+ T$_{VM}$ cell expansion is largely dependent on IL-4. **(A, B)** B6 WT mice were treated with α-GalCer, and serum and spleen were collected at indicated times. IL-4 protein levels in the serum were quantified by ELISA **(A)**. The dotted line depicts the lower limit of quantification, which is the lowest concentration of the standard curve. The mRNA levels of *il4* in the splenocytes were measured by qPCR, normalized to *Gapdh,* and represented as fold change relative to that on day 0 **(B)**. **(C)** Mice were treated with α-GalCer or DMSO and sacrificed 5 days later. Representative flow cytometry plots, percentages, and cell numbers of CD8+ T$_{VM}$ cells in the spleens of B6 WT and CD1d KO mice after α-GalCer treatment. Data are pooled from two independent experiments with a total of five to six mice per group **(A, B)** or two to three independent experiments with a total of seven to ten mice per group **(C)**. Data are

presented as mean ± SEM. $^{**}P < 0.01$; $^{***}P < 0.001$; $^{****}P < 0.0001$ by one-way ANOVA as compared to day 0 control **(A, B)** or one-way ANOVA **(C)**.

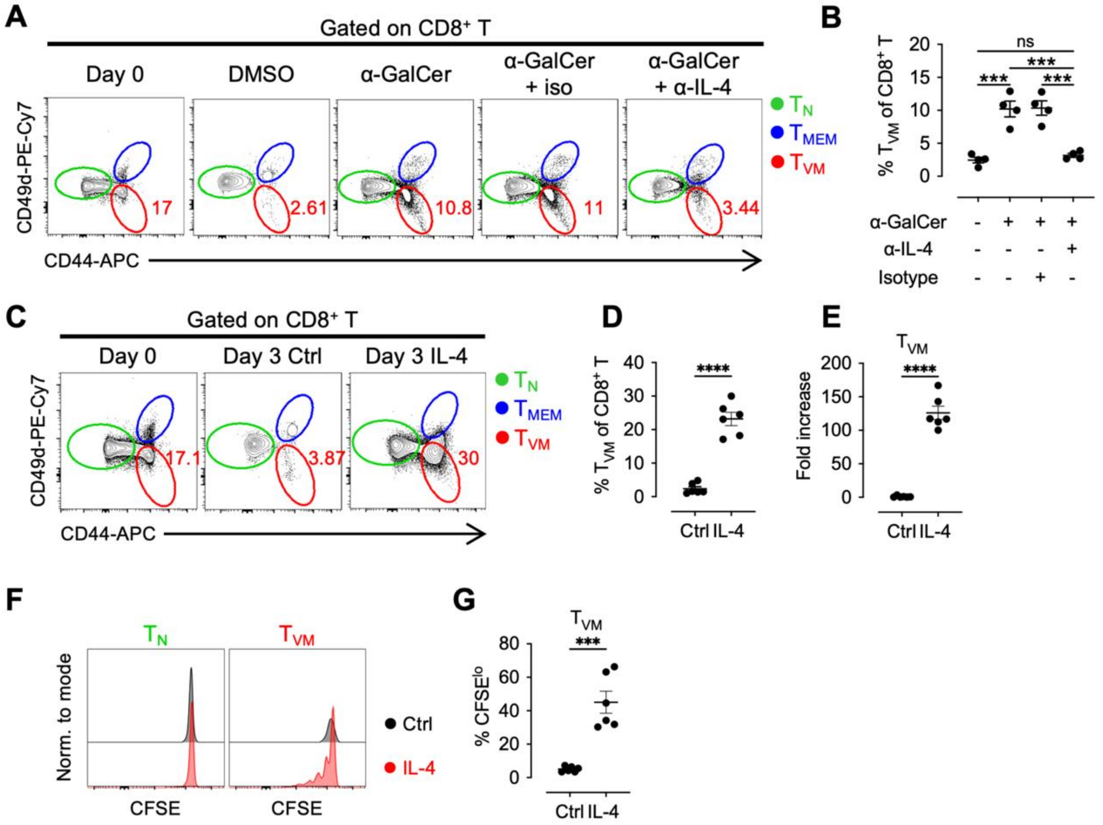

FIGURE S6

IL-4 maintains $CD8^+$ T_{VM} cell population *in vitro* by promoting their proliferation. **(A, B)** Splenocytes harvested from naïve B6 WT mice were cultured in complete RPMI-1640 medium containing DMSO or α-GalCer with or without anti-IL-4 antibody (clone 11B11) or isotype control (rat IgG1, HRPN) for 3 days. Representative flow cytometry plots **(A)** and percentages **(B)** of $CD8^+$ T_{VM} cells in the $CD8^+$ T cell population in different conditions (n=4). **(C-G)** Splenic $CD8^+$ T cells purified from B6 WT mice were labeled with carboxyfluorescein succinimidyl ester (CFSE) and then cultured in complete RPMI-1640 medium or supplemented with recombinant mouse IL-4 for 3 days. Representative flow cytometry plots of $CD8^+$ T cells cultured in different conditions **(C)**. Percentages of $CD8^+$ T_{VM} cells in the $CD8^+$ T cells **(D)**. Viable $CD8^+$ T_{VM} cell numbers normalized to those cultured in complete medium alone and

presented as fold increase **(E)**. Representative flow cytometry histograms of CFSE expression gated on $CD8^+$ T_N and T_{VM} cells **(F)**. $CD8^+$ T_{VM} cells that have lost any level of CFSE labeling were further defined as $CFSE^{lo}$ T_{VM} cells **(G)**. n=6 **(D, E, G)**. Data are presented as mean ± SEM. *P < 0.05; **P < 0.01; ***P < 0.001; ****P < 0.0001 by one-way ANOVA **(B)** or Student's *t* test **(D, E, G)**.

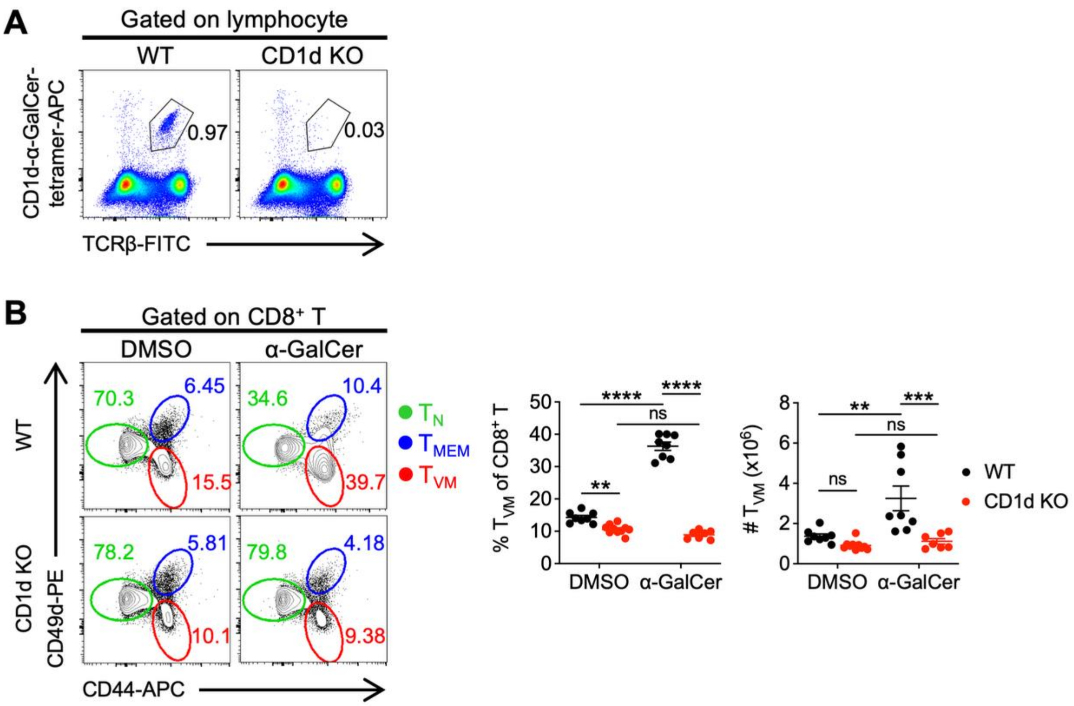

FIGURE S7

α-GalCer-induced CD8+ T_{VM} cell expansion is completely dependent on iNKT cells and the CD1d molecule. B6 WT and CD1d KO mice were treated with α-GalCer or DMSO and sacrificed 5 days later. **(A)** Representative flow cytometry plots of iNKT cells in the spleen of B6 WT and CD1d KO mice. **(B)** Representative flow cytometry plots, percentages, and cell numbers of CD8+ T_{VM} cells in the spleens of B6 WT and CD1d KO mice after α-GalCer treatment. Data are pooled from two to three independent experiments with a total of seven to ten mice per group. Data are presented as mean ± SEM. ns, not significant; $^{**}P < 0.01$; $^{***}P < 0.001$; $^{****}P < 0.0001$ by one-way ANOVA.

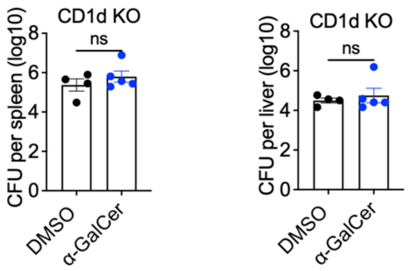

FIGURE S8

α-GalCer treatment does not affect the *Lm* burdens in CD1d KO mice. CD1d KO mice were treated with α-GalCer and infected with *Lm* 14 days after α-GalCer treatment. The spleen and liver were harvested three days after infection. Data are presented as mean ± SEM (n=4). ns, not significant by Student's *t* test.

Table S1. Antibodies used in this study

Specificity	Clone	Format	PRID
**BioLegend**			
CD3ε	145-2C11	FITC	AB_312671
CD3ε	145-2C11	APC-Cy7	AB_1877170
CD3ε	145-2C11	BV605	AB_2565842
CD3ε	145-2C11	BV421	AB_11203705
CD4	GK1.5	BV750	AB_2734150
CD8α	53-6.7	PE-Cy7	AB_312761
CD8α	53-6.7	PE/Dazzle 594	AB_2564027
CD11b	M1/70	APC-Cy7	AB_830642
CD11c	N418	APC	AB_313779
CD44	IM7	APC	AB_312963
CD44	IM7	BV711	AB_2564214
CD49d	R1-2	PE	AB_313039
CD49d	R1-2	PE-Cy7	AB_2563700
CD49d	R1-2	PerCP-Cy5.5	AB_2563702
TCRβ	H57-597	FITC	AB_313429
Ly6G	1A8	BV510	AB_2562937
I-A^b	AF6-120.1	FITC	AB_313725
IFN-γ	XMG1.2	PE	AB_315402
TNF-α	MP6-XT22	FITC	AB_315425
NK1.1	PK136	BV650	AB_2563159

B220	RA3-6B2	PerCP-Cy5.5	AB_893354
TruStain FcX™ (anti-mouse CD16/32)	93	Purified	AB_1574975
**TONBO BioSciences**			
CD45	30-F11	RedFluor 710	AB_2621986
**BioXCell**			
Anti-mouse IL-4	11B11	Purified	AB_1107707
Anti-horseradish peroxidase	HRPN	Purified	AB_1107775
Anti-mouse CD8α	2.43	Purified	AB_1125541
Anti-keyhole limpet hemocyanin	LTF-2	Purified	AB_1107780
**Jackson ImmunoResearch**			
Goat Anti-Mouse IgG + IgM (H+L)	Polyclonal	Purified	AB_2338451
**Tetramer**			
Mouse CD1d PBS-57	-	APC-labeled tetramer	AB_3101905

Table S2. qPCR primer sequences used in this study

Target (mouse)	Primer sequences
Il4	Forward: 5'-TGTCATCCTGCTCTTCTTTCTC-3'<br>Reverse: 5'-TCTGTGGTGTTCTTCGTTGC-3'
Gapdh	Forward: 5'-GTGAGGCCGGTGCTGAGTAT-3'<br>Reverse: 5'-TCATGAGCCCTTCCACAATG-3'
